# Black Women Coaches in Community: Promising Practices for Mentorship in Canada

**DOI:** 10.3389/fspor.2022.884239

**Published:** 2022-04-28

**Authors:** Janelle Joseph, Alex I. McKenzie

**Affiliations:** Indigeneity, Diaspora, Equity, and Anti-racism in Sport (IDEAS) Research Lab, Faculty of Kinesiology and Physical Education, University of Toronto, Toronto, ON, Canada

**Keywords:** mentorship, community, safe space, racialized, intersectionality, sport, coaching, othermothering

## Abstract

Mentorship programs have been shown to help under-represented women navigate their environments, but little research has been done on mentorship programs in sport coaching in Canada. The first of its kind in Canada, the Black Female Coach Mentorship Program (BFCMP) created by the Black Canadian Coaches Association in partnership with the Coaching Association of Canada caters to an historically excluded population: Black, Biracial, and Indigenous women coaches. The research aimed to understand the experiences of program participants to better inform policy, decision-making, and sustainability of the BFCMP. Through mentorship session observations, one-on-one semi-structured interviews with 15 of the 27 inaugural BFCMP mentors and mentees, and thematic analysis, we determined the ability to form a trusted community was a promising practice for coach mentorship programs. Our findings suggest that participants, the majority of whom were the only Black woman coach in their program/institution, benefit from mentorship because of the opportunities to help each other develop as leaders, build relationships to resist loneliness, and nurture resilience through community.

## Introduction

The Black population is growing in Canada, doubling from nearly 600,000 in 1996 to 1.2 million in 2016 (Statistics Canada, 2019). However, this population growth has not coincided with the elimination of economic, social, and health inequities; nor has it necessarily galvanized a considerable increase in data-driven research to address the alarmingly low representation in sport-specific settings at the participant, administrator, or coach level. As such, Black communities are disproportionately affected by social inequalities. Though more than 40% of college and university undergraduates in Canada are estimated to be racialized (Canadian Institute for Health Information, 2020), they account for only 10% of top university positions in sport administration (Heroux and Strashin, 2020). Specifically in Ontario University Athletics, 8.7% of athletes identified as Black but only 5% of coaches and 4.4% of administrators identified as Black, and the majority of those sport leaders were men (Joseph et al., 2021). Black women sport coaches are under-represented.

Little formal data is collected on race and sport in post-secondary institutions and provincial/national sport organizations, particularly due to the white and male dominance of sport in Canada (Joseph et al., 2012) and of sport research in kinesiology (Douglas and Halas, 2013; Nachman et al., 2021). Nevertheless, the research that does exist demonstrates that Black and Indigenous people face discrimination in leadership (Cukier et al., 2020), post-secondary education (Henry and Kobayashi, 2017; Canadian Association of University Teachers, 2018; Nachman et al., 2021), and several institutional reports indicate the problem is pervasive in post-secondary sport (Joseph et al., 2012; University of Toronto Faculty of Kinesiology Physical Education, 2018; Joseph, 2020). With concurrent anti-Black racism and health pandemics permeating many aspects of our lives, the onus to assess and address race-related gaps in Canada has never been more imperative. The miniscule number of Black female coaches is a social inequality issue that requires a structural solution. The most effective and pragmatic way to make informed decisions regarding racial equality is to work collaboratively with communities to gain empirical evidence that can fuel proper policy and decision-making. Most notably, we must collect race-based data (Balkissoon, 2020), with significant attention to assessing anti-racism interventions.

One intervention to redress inequities in leadership and promotion is mentorship programs that help under-represented women navigate their environment (Rhode, 2003), taking into account experiences that directly result from their intersectional identities. This includes, for example, mentorship directed at those living as racialized women, as mothers, with (in)visible disabilities, or as part of the LGBTQ2S+ community (Mansfield and Welton, 2016). With attention to sport, research has shown that Black women need formalized support systems like mentorship to promote and enhance their experience in sport (Francique and Hart, 2010). What remained unknown is how effective these mentorship programs are, especially in Canada. The Black Canadian Coaches Association organized and led the Black Female Coach Mentorship Program (BFCMP) in partnership with the Coaching Association of Canada. This program aims to provide a sustainable model of mentorship focused on accessibility, support, leadership, and coach professionalization (Coaching Association of Canada, 2020b). According to the Sport Information Resource Centre (2020), the BFCMP also produces insights to better inform sport organization action and enhance the participation, mentorship, and leadership of girls and women.

The aim of this study was to partner with the BFCMP to collect qualitative data on who participants are, what they have experienced in sport, and what the program has offered them. We were also interested in their suggestions for improving sport in terms of women and girls participation, racial representation, program adherence, as well as leadership involvement more broadly. Their experiences and suggestions can inform better policy and decision-making, and direct the sustainability of BFCMP and future directions for Coaching Association of Canada mentorship programs. This research is not merely performative equity work (Ahmed, 2006). Instead, it offers effective measures that can spur real anti-racism and gendered change in sporting communities across post-secondary campuses and within recreational, provincial, and national sport.

## Literature Review

### Gender Inequality in Coaching

Inequality in coaching can manifest itself in many ways, and at many different levels in the profession. Defined as “the state of not being equal, especially in status, rights, and opportunities” (United Nations Department of Economic Social Afairs, 2015), inequality in sport has long been commonplace (Heggie, 2020). As an environment historically designed and occupied by cisgendered, heterosexual, white, able-bodied men, individuals who contest these social identities are subject to marginalization within the coaching profession (Norman, 2016; Hammond et al., 2019, 2020; Kempe-Bergman et al., 2020). So far, the majority of research on inequality in coaching has focused on gender inequalities, exploring the representation of and opportunities for women coaches. Despite relatively equal numbers of women in sport and in entry-level coaching education, Norman et al. (2021), Carter-Francique and Olushola (2016), and Reade et al. (2009) found the proportion of women coaches in high-performance and post-secondary sport in assistant and head coaching positions are lower and often stagnant.

Research highlights structural and cultural barriers for women in sport including (a) a lack of financial and material support for women's sport teams—where women coaches are more likely to be employed (Krane and Barber, 2005); (b) cultures that reinforce the notion that women are less capable than men (Kamphoff, 2010); (c) discrimination that causes lesbian coaches to hide their sexuality and personal values for fear of further marginalization (Norman, 2012, 2013, 2016); and (d) a disregard of the existence of gender inequality and the need to increase the representation of women coaches from those positioned to support change (Kempe-Bergman et al., 2020). Furthermore, research examining motherhood in coaching points to inadequate administrative and structural resources to help support women with families and children advance or remain in coaching roles (Theberge, 1992; Kamphoff, 2010; Bruening et al., 2016). Often expressing having to choose between career goals and starting/maintaining a family, women coaches frequently face discrimination in the profession.

Discrimination can be perpetuated both on an individual level, such as a lack of respect or concern, for example, and on a structural level, such as the slowing of career progress, earning capacity, and privileges afforded to male coaches and teams. For instance, lesbian coaches can experience an inability to recruit athletes, loss of jobs, and limited professional mobility (Krane and Barber, 2005). Both forms of discrimination serve to create and maintain barriers in coaching and continue to perpetuate narratives that coaching is a space exclusively for men (Kamphoff, 2010; Bruening et al., 2016) and further supports the false ideological assumption that men are superior leaders (Norman, 2012). Thus, strategies to improve gender equality in coaching must address individual and structural barriers.

### Racial Inequality in Coaching

Canada has gained a global reputation as one of the most diverse, inclusive, and progressive nations (Greenhill and Marshall, 2016). As the first country to codify a Multiculturalism Act (Srikanth, 2012), and with rising immigration (Statistics Canada, 2019), Canada is broadly depicted as an inclusive society, and thus, inoculated to racism, racial injustice, and racial inequities. The result is a state of “democratic racism”—a concept Henry et al. (2010) use to describe how Canadians can simultaneously hold denigrating ideas about racialized populations that maintain harmful systems and structures, while employing libertarian ethics such as equality, justice, and tolerance.

Racism has historically been weaponized in sport (Cunningham, 2020; Cunningham et al., 2021). This is particularly discernable in Canada (Joseph et al., 2012; Forsyth and Giles, 2013), leaving many racialized and Indigenous communities bearing the brunt of its institutional implications. Racism permeates many aspects of sport, from the positions racialized athletes play (Sack et al., 2005; Cunningham, 2010), to the coaching and administration positions racialized stakeholders can secure (Anderson, 1993; McDowell and Cunningham, 2007). Racist ideologies, including White athletes (and coaches) being smarter and more ethical than Blacks (Coakley, 2009), impinges upon Black sport participants. Though institutional racism represents a macro-level factor impacting Black coaches and athletes, there are also meso-level (i.e., prejudice and discrimination; Cunningham, 2010), and micro-level (i.e., high staff turnover; Cunningham et al., 2019) variables blocking Black career opportunity and advancement in sport coaching. The outcome is an under-representation of Black head coaches and decision-makers at all levels of sport, whether grassroot, developmental, or high-performance.

Racially and ethnically marginalized groups are disproportionately under-represented in sport as athletes (Lapchick et al., 2010; Acosta and Carpenter, 2012; Joseph et al., 2021) and as decision-makers (i.e., coaches, athletic directors; Lapchick et al., 2010; Carter-Francique and Olushola, 2016; Joseph et al., 2021). Regarding racialized girls and women, Carter-Francique and Flowers (2013) propose that numerous factors—historical, economical, social, religious, and legal—can contribute to under-representation. They advance that comprehending these variables, and how they interact with racism and sexism, is necessary to achieve the full benefits of sport for all. Racism and sexism have led to the othering of racialized girls and women, which has limited access and opportunities in sport (Hylton, 2018). Carter-Francique and Olushola (2016) indicate that enacting more race-based and gender-based legislation—because gender and race cannot be detached—can improve sport. Still, there is scarce research and scant support systems offering insight into the successful pathways enabling the growth and promotion of racialized women in coaching (Carter-Francique and Olushola, 2016). This indicates a demand for more research and resources geared toward supporting the entry and advancement of racialized women in coaching.

### Reforming Inequalities in Coaching Through Mentorship

Representation of women in coaching has been examined, with most studies specifically addressing gender inequality without attention to race (Callary, 2012; Culver et al., 2019; Kraft et al., 2020). Women in coaching have demanded transformational change to factors contributing to the dearth of women coaches in the workforce, including (a) the gendering of coaching as masculine; (b) exclusion from coaching networks; (c) lack of support from governing bodies; and (d) discriminatory structural practices and cultural attitudes (West et al., 2001; Norman, 2008; Cunningham et al., 2019). Where coaching is traditionally seen as an “Old Boy's Club” (Coaching Association of Canada, 2020a), it is further detailed as an “Old White Boy's Club” in Canadian university athletics (Joseph et al., 2021). Increasing racial representation among coaches has been acknowledged as a strategy to reform coaching inequalities, along with transforming racist cultures through education and policy change (Joseph et al., 2021). White men coaches are invited into and participate in (in)formal coaching networks and mentorship opportunities, where they are put into contact with decision-makers for recruitment, especially for head coaching positions (West et al., 2001; Norman, 2008; Cunningham et al., 2019, 2021). The exclusionary networks coinciding with non-inclusive and unsupportive work environments and policies contribute to the onslaught of occupational turnover and under-representation of women coaches (Cunningham et al., 2019; Nesseler et al., 2021) and racialized coaches (Bradbury, 2013; Rankin-Wright et al., 2017, 2019; Bradbury et al., 2018; Cunningham, 2020; Rankin-Wright and Hylton, 2020).

Though some women coaches—and more specifically racialized coaches—have reported more athletic and coaching experience relative to their White male counterparts, they are still overlooked and undervalued for senior coaching positions in high-performance sport (Larsen and Clayton, 2019). Based on the experiences of women coaches, increasing opportunities for mentorship and support is a prominent and effective recommendation for helping to support women and bolster their representation and recognition as valued members in coaching. Women coaches who have formal and informal mentoring relationships during all stages of their career were more likely to continue in coaching, and progress to a higher level (Banwell et al., 2019; Wasend and LaVoi, 2019; Norman et al., 2021).

Outside of career-specific mentorship, Banwell et al. (2019) found that women coaches also require acceptance, friendship, role modeling, counseling, and confirmation from the support networks created through mentoring. They recommend that this come in concert with mentoring from coaches both internal and external to their sport organizations. These additional types of support systems help expose women coaches to new professional development and job opportunities while fortifying confidence and self-efficacy, positioning them to better prosper and maintain their roles in coaching. Providing sponsorship—a person to speak on behalf of a coach when she is not in the room—along with mentorship to women coaches helps mitigate the structural barriers that confront women in coaching, and enables a more “level playing field” compared to simply increasing the number of women represented in coaching positions (Norman, 2008). Increasing the number of women within a misogynist environment does not solve the problem of gender inequality in coaching.

To nurture the next generation of athletic leaders, women in Borland and Bruening'ss (2010) study became “adamant about the importance of reaching back and helping student-athletes and young girls in their communities in an effort to establish a tradition of adult Black females helping young Black females” (p. 415). These tactics of building Black confidence through mentorship, sharing an ethic of care, advocacy through a maternal style, and guidance related to survival, have been explored extensively as “othermothering”. Originally espoused in the early 1990s as pedagogical practices in support of Black children and students (Collins, 1991; Henry, 1992; Foster, 1993), othermothering has been more recently elaborated by activist-scholars as a “politicized ethic of care” in education (Lane, 2018; Watson, 2018; McArthur and Lane, 2019). Black politicized educators emotionally invest in others “through an authentic form of caring that includes a love for self and community” (Lane, 2018, p. 276). Othermothering has been shown to contribute to community building among Black women, in the United States in both elementary education (Foster, 1993; Loder, 2005) and post-secondary education (Hirt et al., 2008; Njoku et al., 2017), as well as in Canada through institutional change (Bernard et al., 2013) and student/teacher learning (Bernard et al., 2000). The utility of othermothering in sport coaching has not been explored.

Building community among Black women in sport is precisely what the present study aimed to investigate, as there is no research on mentorship programs for racialized women coaches in Canada. The research was designed to investigate: How do intersectional factors of race and gender influence sport coaching? How do Black women coaches in Canada experience this mentorship program? What might their experiences tell us about mentorship program value and sustainability?

## Materials and Methods

The second author conducted observations of three mentorship sessions and facilitated 15 one-on-one interviews with nine mentors and six mentees in the inaugural BFCMP. All sessions and interviews were online via Zoom. Sessions were not recorded, but field notes were written in a notebook to document participant verbal and gestural interactions, reactions, and major themes discussed. All interviews were recorded using Zoom, which were then transcribed through Otter.ai to create verbatim transcripts. Using thematic analysis, transcribed data was divided into meaningful pieces of information called meaning units. Both authors compared and grouped meaning units into distinct categories in a process known as “creating categories” (Côté and Salmela, 1994). These categories emerged as themes and subthemes that were coded. Research Ethics Approval was acquired from the University of Toronto Research Ethics Board (Protocol # 40567).

### Participant Observation

Participant observation is a process that affords researchers an ability to learn about the experiences and activities of participants of interest in their natural setting through examination and engagement in that environment “to develop a holistic understanding of the phenomena under study that is as objective and accurate as possible” (Musante and DeWalt, 2010, p. 110). This style of research is typified by being an attentive listener and observer able to adapt to the unexpected, and by having an openness to others while also being inquisitive in learning more about them (DeWalt and DeWalt, 1998). As such, this methodology is an applicable approach for answering descriptive research questions. This unstructured method of observation is grounded in interpretivist and constructivist approaches extending beyond positivism. Emphasizing “the importance of context and the co-construction of knowledge between researcher and ‘researched”' (Mulhall, 2003, p. 306), unstructured approaches foster better comprehension of participant experiences.

In our study, observation was designed to monitor the BFCMP mentorship program registration, adherence, and participation rates. It was also used to collect evidence about promising practices for success based on participants' unprompted comments in their online meetings. The researcher introduced himself in every session, kept his camera on, but did not otherwise interrupt the session.

### Interviews

As the most commonly used data collection device in qualitative research (Sandelowski, 2002), one-on-one interviews are known as the “gold standard” for qualitative data collection (Silverman, 2000, p. 291). Specific to the current research, one-on-one semi-structured interviews involved a hybrid approach with open- and close-ended questions, typically followed with probing *whys* and *hows*, following the guidance of Newcomer et al. (2015, p. 366). This strategy offers more opportunities for organic conversation that also creates room for exploring a wide range of (un)intended topics. The interview guide was designed to inquire about participants' past experiences with respect to race and gender in coaching; the ways participating in the mentorship program changed their behaviors, thoughts, and feelings; their most and least preferred aspects of the program; and their recommendations for future mentorship programs in sport coaching.

#### Participants

Interview participants were recruited directly from the program and all self-identified as women and as Black, Biracial, or Indigenous. These racial categories are neither exclusive nor static. A rigorous application process to enter the BFCMP assured that participants all had coaching experience, and were currently obtaining or pursuing a “Registered Coach license” or their “Chartered Professional Coach designation.” The participants were between the ages of 21 and 60, with primary coaching experience in basketball (*n* = 9). Other sports coached included track and field, volleyball, ice hockey, swimming, and rugby (exact numbers of mentors and mentees in each sport are withheld to protect participant anonymity). They coached in community and recreational sport, university and college varsity sport, and within provincial/national sport systems at varying times. Mentors completed the National Coaching Certification Program Mentorship prior to, or at the beginning of the BFCMP mentorship program. Mentors had monthly meetings referred to as “check-ins” with each other, and were expected to communicate regularly with their assigned mentee, meeting at least once per month.

## Results

Participants' mentorship sessions and interviews revealed that the program played a significant role in their feelings of belonging and support in the field of coaching. Mentorship was important in three main domains: (1) Helping and Developing Other Leaders, (2) Building Relationships to Resist Loneliness, and (3) Nurturing Resilience through Community. These themes and the ways they contribute to creating a networked community of Black women coaches are described below.

### Helping and Developing Other Leaders

Participants described mentorship as important because it is part of their intergenerational legacy. Mentors felt a sense of responsibility to build community and help others who are more junior and in need of the transmission of culture, traditions, wisdom, and care. Sharon and Sarah expressed a sense of joy related to the responsibility to help others:

Definitely the biggest joy … it's meeting people, helping people, watching people grow. And that's kind of why I wanted to be involved. (Sharon, basketball, mentor)I didn't recognize really at the time, when I was younger, that [coaches] *were* my mentors and *were* leaders to me … [they had] a profound positive effect … being in that [mentor] position and knowing the responsibility that I have and how helpful I can be to them in any way, in whatever capacity, that is extremely rewarding. (Sarah, hockey, mentor)

Until participating in the BFCMP, some coaches hadn't named the help they had provided previously as mentorship, and were unaware of how much others could benefit from hearing about their accomplishments, experiences, and problem-solving strategies. Putting the label of mentorship on their process allowed them to solidify their commitment to educating others and clarify the learning outcomes they wanted to convey related to the often hidden culture of role models in coaching.

I didn't realize how they view me in high regards. Maybe I didn't view myself like that … I just do what I do. … So when I'm speaking with my mentees and … they say … the things I helped them with, I was like wow that actually kind of made me realize, OK, I guess I do have something to give. (Della, basketball, mentor)

I'd spent so much time focusing on my program and surviving my coaching experience that I wasn't really thinking of putting myself out there from a national perspective in terms of mentor[ing]. But this program has definitely helped me understand that I have a responsibility for the next generation of coaches, and that it is important that they see someone thriving as a Black coach that's experienced some of the same challenges … they are currently going through. [I help them understand] they can survive those things and they can thrive and they can be at the highest level in our country and I don't think it's as impossible as it maybe was even a few years ago. … [Other] coaches don't always understand the struggle of their athletes simply because they just don't have that [racial and gender] perspective. (Chaya, basketball, mentor)

Chaya participated in a longstanding tradition of Black women's sense of responsibility to work in “caring for their communities …[which] served for many as an expression of political activism” (Collins, 2019, p. 168). Through her presence and storytelling, she was able to empower other women in a system that historically excluded them, which allowed them to better support racialized women athletes as well as each other.

Della became clear that mentoring coaches is about helping them to develop much more than technical skills:

It's not just about coaching [techniques] … It was about developing their skills and what they're going to need to coach. … If you don't develop those leadership skills, right, then you're not going to be successful. So we did a lot on that type of stuff … working on communication, teamwork … self-care, self-love, things like that. All those skills within leadership were demonstrated through that, so it was good to help them with that as well. (Della, basketball, mentor)

Self-care and self-love are two essential leadership skills for Black women coaches to understand and be able to communicate to their athletes. Self-care is “not self-indulgence”, but a radical “act of political warfare” (Lorde, 1988, p. 125) in spaces that marginalize and exclude women leaders and racialized experts. There are so many demands put on Black women that they become prone to burnout. As such, learning to take care of oneself is a key aspect of coach retention.

Leadership in coaching requires Black women to share what they know, building each other up before they can “take on these spaces,” as Jolene puts it:

[Coaching is] a lot more about leadership than I initially had thought and even though I'm young and I'm a young leader, I still can teach those around me a lot and give them a lot of resources that they can use and give them a sense of community … and confidence that they need to take on these spaces that weren't made for us. (Jolene, rugby, mentor)

Jolene helped her mentee by providing education about how to navigate mainly White and mainly men's sport spaces, coaching and personal development resources, access to a community network, and by building confidence through reinforcing what her mentee already knows and does. Her othermothering tactics demonstrate the importance of Black feminist traditions that emphasize the “sociopolitical and affective aspects of teaching (e.g., race-gender hierarchies, love, and trust)” (Lane, 2018, p. 275). The quotes above from mentors and mentees in the BFCMP show that in coach mentoring, as in education and community organizing, a Black woman mentor “goes beyond the institutional goal of solely promoting cognitive growth and focuses on the socioemotional well-being of Black [mentees, based on] understanding the[ir] marginalization and invisibility” (Greene, 2020, p. 2). In other words, helping and developing leaders requires recognition of the context in which they will ultimately work and the specific skills they will need to navigate those spaces.

### Building Relationships to Resist Loneliness

Many of the BFCMP mentors imparted in their mentees the importance of building relationships to resist loneliness, feeling like a fraud, or sensing exclusion. Reaching out to strangers to develop networks can be challenging, nerve wracking, and time consuming, but many coach mentors identified their valuable capacity to expand and grow the networks of mentees through direct referrals. According to Sharon, basketball coach and mentor, developing a network takes “months” or “years,” but “just having a referral or [introduction] was a big help for [my mentee]. So that was one of the highlights for me.” Being able to provide access to their networks so that mentees could establish their own relationships allowed mentors to feel useful, especially as they recognized relationship building as a key element of their own access to training, jobs, and overall success as coaches.

The deeper they were able to build trust and fun with their mentees, the more mentors realized their role not as a person to teach coaching techniques, but as an empathetic sounding board for mentees to share their ideas and experiences, thereby enhancing feelings of being valued and understood.

Getting to work with my mentee was a lot of fun. We had a good time together. … There wasn't a whole lot of coaching [discussions] … but we ended up talking a lot about what her other goals were … We just built a really good relationship and it was just kind of fun to see somebody figure out the bits and pieces of what she wanted to do. (Chamanda, swimming, mentor)

This program has been great in so many ways where it's just not about the Xs and Os. But you know, sometimes needing a familiar voice or things to navigate these unique situations, and to be an ear or a person not to judge, but just to listen and give advice. And that's why I'm very appreciative … [that] a relationship is built out of this mentorship program. (Nika, basketball, mentee)

The “Xs and Os” refers to the techniques of coaching sports such as basketball, football, hockey or soccer, where coaches draw offensive and defensive plays. Nika is clear that what she learned from her mentor went far beyond in-game strategy. She gained non-judgemental advice from a trusted colleague, which was really important because she didn't feel she had someone in her organization with whom she could meaningfully converse.

Having a deep connection allowed BFCMP participants to learn from one another. Research demonstrates that when Black women can relax, and share honest stories, exchange humor, and compare experiences with each other, they counter everyday processes of dehumanization and create a “homeplace” (Goins, 2011), while they “pierce the invisibility created by Black women's objectification” (Collins, 2000, p. 104). Mentees were open and vulnerable, sharing personal and professional stories that allowed them to understand that others went through what they were going through.

[I] don't feel as alone anymore because, you know, going through, trying to navigate my career … [in] not just a predominantly male industry, but also the females are always – they're mostly white, right? It really, really hasn't been easy. … [Now, if] I have issues I know I have people that I can call on. (Nora, basketball, mentee)

The best part of the program…I loved connecting with other Black female coaches. … Just to see what they're doing, and then also to hear some of the struggles that they have, which kind of made me [say] “Wow, I struggle with that too! Like, I thought it was just me!” … It was good to hear that and then how they would tackle it. (Della, basketball, mentor)

Nearly every participant described resisting being alone, social isolation, feeling lonely, or believing they were the only one with their experience as a key benefit of the program. Nora, basketball coach and mentee, said “This program was effective …[because] I feel [support in] anything that comes my way … I don't feel as alone anymore.” Similarly Ariel, volleyball coach and mentee, described relishing “the opportunity to connect with some amazing Black females and come to realize that we all go through some similar struggles.” And Nika, basketball coach and mentee, also shared “I realize that a lot of things that I have been feeling… everybody that was a part of that group has felt … the same way.” Being able to talk to coaches who look like them normalized their experiences.

Within the framework of a Black mentorship program, Sarah was so happy to learn that her mentee was also African and committed to long term support beyond the program.

You feel more comfortable when you're talking with someone … [and] you have that common ground, so it was so great. To speak to someone who is passionate about sport, who is passionate about helping others. I just felt I wanted to help her so much in any way possible and just be a constant support for her and tell her that I'm always here and I'm going to check on you. You know, six months, nine months down the road and continue to check on you because I know for me, if I had that when I was her age, I would have probably taken that next step … to get into coaching. (Sarah, hockey, mentor)

Sarah expressed here what Lane (2018) refers to as “love,” a politicized ethic of care and accountability similar to a familial mother-daughter or sister-sister relationship, with high levels of commitment to and future expectations for a mentee whom she will “check on”. Ariel, volleyball coach and mentee, also described building relationships beyond professional and into the realm of friendship and is “so grateful for this opportunity to network and make lifelong friends.” A commitment to staying in contact beyond the program was a direct strategy to combat loneliness in the field.

In sport settings where white supremacy and cis-heteropatriarchy are overarching ideologies accompanying the limited number of Black women coaches, they experience isolation, undercompensation, microaggressions, marginalization, and tokenism. Chaya mentioned there is an emotional toll of coaching-while-Black as a woman.

We're celebrating having people in leadership roles that are women and people of color, which is great. But then the isolation that they feel, or the responsibility to represent all people of color … and then [being] expected to speak into every [equity] situation all the time … becomes emotionally exhausting. (Chaya, basketball, mentor)

Emotional support is a key function of the BCFMP. “As a coping mechanism,” Minnet et al. (2019, p. 215) note, “Black women's friendships function as sites of empowerment and resistance from oppression,” as women offer each other affirmation, a safe space/homeplace, support, and encouragement within racist and sexist environments.

### Nurturing Resilience Through Community

For historically excluded groups such as Black women in sport coaching, a sense of community can harness feelings of belonging, motivation, optimism and resilience. For Black women coaches who are the only ones in their predominantly white institutions, a sense of community must be intentionally built beyond the boundaries of their organizations. Jolene described learning from other women in other sports in other provinces how to navigate isolating spaces, and learning to be her true self, thus building resilience.

[Coaching is] kind of tough to navigate because you don't have a sense of community or a sense of belonging. … The Black female coach mentorship program, it's given me a sense of community of [how] people are going through and navigating these same spaces that I'm navigating. … It was a great sense of community. (Jolene, rugby, mentor)

In Clarissa's case, the need for community and a safe space to reflect on the challenges she had faced came as a surprise.

I changed [my] feelings around the importance of creating groups and spaces like that. I never had a female mentor that was Black or not White [before]. I didn't know I had a need for places like that. … I remember the first time mentors did our course together, five or six hours of talking you realize how much shared experiences you have. We are all high level coaches so we know the grind and what it takes to be a winner but didn't know how important it was to reflect. (Clarrisa, basketball, mentor)

The “grind” is more than the struggles related to enhancing sport performance in athletes. It is related to remaining steadfast in a system where racism and sexism are prominent. Chaya and Tiffany note that the “grind” requires resilience. While survival is individual, resilience is collective. These findings confirm what Rich et al. (2021) suggest are two of the important ways community has been explored in relation to sport. Community is an outcome that can be built or strengthened in and through sport via relationships and networks that allow for the development of social capital. Community is also a site for resistance in sport, whereby groups that have historically faced discrimination and disadvantage mobilize to enhance inclusivity. Building a community together means Black women can collectively see each other being successful in the face of struggles. This means any one of them can live through the hassles of their work and still achieve greatness, changing their institutions in the process. Both women note that their institutions will not create community for them.

I wouldn't necessarily say that the organizations that we're part of are helping us … [build] a community…. We're spending so much time trying to survive that it's hard to put your head up sometimes to see who else is out there, and I think that's what this COVID year has actually really supported is realizing that there are a lot of people that are wrestling and struggling with the exact same things that we are. And now we have a responsibility to make sure that we build that community that we need so that we can all see ourselves, you know, succeeding within our context. (Chaya, basketball, mentor)

[I] stand more firmly in my beliefs that change needs to occur urgently. Of course, it is nice to feel connected to community, but it's frustrating to know many of us have had the same or similar [negative] experiences. That being said, this [mentorship program] gave me a sense of optimism that we have a strong community and a bright future. (Tiffany, basketball, mentor)

Jolene, rugby coach and mentor, emphasized that because Black women have traditionally been left out of head coach positions, they have to “put on a front … in a space that wasn't created for you, that never even thought of you to begin with,” that is, in sport institutions led mostly by White men. Collins (2000, p. 97) refers to this front as a “mask of behavioral conformity” imposed on Black women that demands individual and collective acts of resistance. In the BFCMP, Black women like Jolene resisted through creating their own safe setting, “a place you can confide in and be your true self in. You don't have to put on a front. You don't have to … put on that White, acceptable facade in order to be accepted.” Because Jolene could be her “true self” she left with an understanding of the need to create her own self-definitions “within social spaces where Black women speak freely…a necessary condition for Black women's resistance” (Collins, 2000, p. 111). She left with an enhanced sense of community and belonging.

A sub-group of the program was designed for mentors only. The objective was to discuss strategies to support their mentees; however, the sub-group ended up being an essential resource for more senior coaches to find support within their own communities.

It was so nice to have, from the mentor side, to have that little community of so many other Black coaches. That was lovely 'cause it's something that I definitely don't experience. I've always been [in] a sport that's pretty much entirely White faces. … As a Black woman when you're in so many White spaces, it's just, it's just the norm. That's your day to day until you get to a space where you're no longer the only Black face. … All of a sudden it's a new level of comfort that you don't even realize you were missing. (Chamanada, swimming, mentor)

Mentor coaches shared their gratitude for the support they had received, and their intentions to maintain that community after the program ended.

The community piece is definitely a big part of it, especially with the challenges that COVID has created for this season. To have a community of coaches across the country really kind of come together to support each other and when we kind of went back and forth about whether we were going to have continued mentor calls, just mentor-only calls. It was a definite yes. We're going to keep doing this because I think we needed that community as much as the younger coaches. (Chaya, basketball, mentor)

The community helped them to understand that their success required challenging the status quo and specific skill-building.

I didn't believe that I could be a coach because of lack of representation in my community. … [Black women] belong in the sport world but there wasn't a community that recognized us or encouraged us. … White coaches naturally have mentors. What about us?… [Who can] be the mentor you need to ensure you get your certification … [or go to] coach clinics?… Knowing that there is a BIPOC community that is there if I need their support … I'm not as alone, or isolated. (Fiona, basketball, mentee)

Participation in the BFCMP has clear benefits for helping participants feel supported, know where to go for information, and be connected to people who share some of their experiences. Although coaching is often considered an individual job, every coach can benefit from having a network to rely on.

## Discussion

This study offers several theoretical insights and practical recommendations for coach mentorship. From a theoretical perspective, intersectional identities of coaches are essential to consider if the aim is to improve coach retention and progression. Crenshaw (1989) is credited with coining the term intersectionality, and drawing attention to barriers Black women specifically face. Black women are often overlooked in programming or policy designed to address race or gender. Considering race and gender together offers additional insights, particularly in Canadian sport that is white and male dominated. The concept of intersectionality has recently been elaborated to consider the ways it is situated within a decolonial knowledge project that demands critical theorizing, examinations of power, and validates the experiences of people from a wide array of social groups (Collins, 2019), in person and online.

Specific to this project, intersectional knowledges were validated through virtual interactions. Noble and Tynes (2016) outline how online spaces have enabled conversations, friendships, and solidarities that used to take place in person, to move into virtual worlds. In a nation as large as Canada, online spaces allow for people to connect across disparate geographies. This is particularly important in a Black diaspora sport setting where Canadians regularly cross borders for social and professional networks essential for their survival in cities rife with interpersonal and systemic racism (Joseph, 2017). Based on ethnographic research with traveling Black cricket players and spectators, Joseph (2017, p. 13) provides an analysis that is equally applicable to Black coaches who connect virtually: “In Canada, black aspirations for belonging are activated by specific local debates about the exclusivity of Canadian national white belonging, and also by desires for membership in an inclusive community that happens to be dispersed across borders.” Connecting across disparate geographies through mentorship to intentionally bolster coaching leadership skills, build relationships, and nurture resilience through community is an anti-hegemonic, intersectional cultural practice in Black communities.

Mentorship proved to be essential for Black women coaches, most of whom were “the only” Black woman at their institution/organization. Their identities as mothers, as novices, as leaders, and other categories influenced their need for mentorship and their experiences as coaches. While an online mentorship program provided access to the support and validation they needed, we must continue to ask how the construction of community may be experienced differently by different folks in [sport] organizations (Rich et al., 2021, Critically Assessing Community, para 8). Race and gender were important to all participants, but their oppression and successes were related to the complexity of their whole selves.

Theoretically, this study also reinforces the importance of critical race theory as a necessary paradigm in Canadian sport studies. Solórzano and Yosso (2002) describe critical race theory as aiming to offer “a liberatory or transformative solution to racial, gender, and class subordination” (p. 24). Racialized coaches experience sport through a lens of social inequities and “everyday racism” (Essed, 1991; Este et al., 2018; Joseph et al., 2021). Racism and the attendant white privilege that are sustained in Canada as an ongoing aspect of everyday life are exposed through critical race theory, which also demands that we seek solutions to this intractable problem (Joseph et al., 2012).

Importantly, Black women coaches in the mentorship program learn from each other that one of the ways to combat racism is to be successful in their positions and rise through the ranks of coaching to be the representation that they were unable to see as athletes and junior/assistant coaches. Moreover, the program gave them an opportunity to reflect on their experiences and talent, develop their knowledge of sport and of self, explore self-love, and learn how to speak up, all of which will ultimately result in coaching and life success. Critical race theory demands research centered around the knowledge and experiences of racialized people, seeing both as “sources of strength” that result in better comprehension of the issue (Solórzano and Yosso, 2002, p. 24). This paradigm has proven effective in understanding the experiences of BFCMP participants. Though racism is a permanent feature of the sport settings they are working in, they have many strategies in place to address their intersectional oppression and to bring anti-racist, anti-sexist agendas to sport in Canada.

Specifically, some of the strategies they use are particular to Black women's communities, for example, “othermothering.” The concept of “othermothering” drawn from traditional Black knowledge practices, is useful to conceptualize the actions of coach mentors who exhibit a sense of responsibility to care for and educate mentees. Because Black women coaches in Canada have mainly not had the benefit of Black women mentors, they have not been able to benefit from the wisdom of other, more senior, and more experienced Black women in their field. Yet in Black traditional oral cultures, women would expect to learn from their elders and ancestors in casual daily settings. Borland and Bruening (2010) found that Black women coaches used phrases such as “pay it back,” and “each one, teach one,” demonstrating a relationship between receiving mentorship and desiring to become a mentor, sharing knowledge with the next generation and planting the seeds for intergenerational relationships to continue. This study extends current research on othermothering by providing a sport coaching example of the heavy emphasis on a “politicized ethic of care” that has been well-demonstrated among Black women teachers and community leaders (Lane, 2018; Watson, 2018; McArthur and Lane, 2019). Othermothering is demonstrated by Black women coaches in Canada who work on behalf of the Black community and thrive in developing the leadership success of their peers.

Othermothering is rarely done alone. Coaches gain emotional strength and resilience, and empower themselves through practices of collectivity. Lorde (1984) reminds readers of the ways:

Black women have always bonded together in support of each other …We need only look at the close, although highly complex and involved, relationships between African co-wives, or at the Amazon warriors of ancient Dahomey who fought together … when we enjoyed each other in a sisterhood of work and play and power” (p. 49–50).

The idea of strong Black women's friendships, a “sisterhood”, as core to survival and to the political acts necessary to change oppressive systems is not new. Black women's radical friendships have been explored within community work as essential components of racial politics and feminist thought. Black women's labor in service of fighting for freedom and social justice has always taken place in the comfort of daily conversations that generate what Collins (2000, p. 103) refers to as a “shared recognition … among women who do not know one another but who see the need to value Black womanhood.” Collins (2000, p. 104) asks, “if we will not listen to one another, then who will?” Talking, listening, sharing, understanding and being understood were key to the relationship development that formed one of the main outcomes of the mentorship program. Drawing from their data and important research on Black women's friendship (e.g., Collins, 2000; Goins, 2011; Bryant-Davis, 2013) Minnet et al. (2019) conclude that because Black women share similar life experiences that are often plagued with oppression and/or trauma, the comfort and collective freedom they find with each other allows their friendships to flourish over the long term. Similarly, BFCMP participants committed explicitly to lifelong friendships and to checking in with each other after the program ended.

In practical terms, this study demonstrates the many ways community is mobilized by Black women for their wellness and skill development. All coaches need mentors to promote career mobility and personal growth (Wright and Smith, 2000; Pastore, 2003; Jones et al., 2009). The results of this study show that mentees and mentors connected with each other to advance their goals and having people who look like them was paramount in enhancing comfort, reducing the necessity of explaining experiences such as micro-aggressions, and developing a social network of people with similar experiences. Mentorship has been shown to be a valuable tool to support student-athletes who experience a lack of racial representation among student-athletes and coaches, as well as athletic and institutional leadership at historically white post-secondary institutions (Brandon, 2012; Bimper, 2017). The findings of this study echo previous research that demonstrated mentorship effectiveness in improving retention of Black women (Abney, 1988, 2007; Abney and Richey, 1991). Abney (1988) noted that a lack of mentors and role models made it difficult for future generations to see a career in athletic administration or coaching as attainable. More recently, Borland and Bruening (2010) found that Black women who were under-represented in head coaching jobs in Division 1 women's basketball in the United States discussed the importance of accessing and cultivating important social networks, seeing role models, having mentors, and development programs as key to retention and promotion. Participants identified that “unless a mentor approaches them or they approach a mentor, these women are apt to feel cut off in athletic departments …because they are sometimes the only Black woman” (Borland and Bruening, 2010, p. 414). The lack of diversity in coaching and limited promotion opportunities for Black women are particularly concerning as head coaching jobs are often precursors to athletic leadership positions and, consequently, Black community wealth. As a result of this study, findings from the U.S. about Black women coaches' treatment discrimination and need for networking are now replicated in Canada.

Community is built one relationship at a time, and this study shows that formal mentorship programs can facilitate the relationships Black women seek and need. Akin to Carter-Francique and Olushola (2016), our findings highlight that structural supports such as training, networking, and advocacy among Black women coaches are fundamental to their success inside and outside of sport. However, their research was bound to a U.S. perspective and did not focus on actively fostering relationships through mentorship. The current results demonstrate that decision-makers in Canadian sport should provide opportunities for intergenerational and peer-to-peer mentorship to occur. Otherwise, Black women's knowledge is silenced and their bodies become invisible from the levers of power. Nzindukiyimana and Wamsley's (2019) study demonstrates that in Canada specifically, few Black women's sport histories are preserved in public archives and written records, yet women have been involved in sport including sport coaching for decades. To resist the silencing and exclusion of Black women in sport, concrete strategies must be put in place to enhance their presence, retention, learning and community building, and more research is needed to share their stories of resilience and development through mentorship.

## Limitations and Future Extensions

Due to Canadian COVID-19 related restrictions, mentors and mentees were unable to meet in person for their learning sessions or one-on-one meetings and as researchers we were unable to fully immerse ourselves into the research environment. Future extensions should consider the advantages in-person research can have in building rapport with and among participants, and on robust participant observation of mentorship sessions, which may reveal interactions not observed in a virtual environment. Further, future research on Black women coach mentorship should aim for a greater number of sessions to observe and people to interview to ensure a broader representation of sports (the majority were from basketball) and coaches at different levels to even better clarify the wide range of experiences of coaches and benefits/drawbacks to mentorship programs. Though the number of Black women coaches outside of the most populous province, Ontario, may pale in comparison, their experiences are no less significant. Moreover, future research in community sport could be used to contrast the experiences of the high performance coaches studied here. Research on Black women coaches should focus on expanding the participant pool so that it better reflects the Canadian experience. Last, within Black women's sport literature, attention to issues related to race and gender such as bi-racial or disabled identities, diasporic or transnational links, and gender fluidity or trans identities are essential as analyses around these identity factors in Canadian settings is nearly non-existent.

## Conclusion

This study adds to the nascent literature that extolls the benefits of mentorship for Black women in higher education (Crawford and Smith, 2005), business (Bova, 2000), and sport coaching (Abney, 1988, 2007; Abney and Richey, 1991), all of which focus on African American examples. This study provides a Canadian example of the Black Female Coach Mentorship Program and shows that mentorship is a key strategy for empowering Black women coaches. A coach mentorship program that focuses on a group with shared identities can offer a concrete method for developing leadership skills, combating loneliness, and building resilience through community.

## Data Availability Statement

The raw data supporting the conclusions of this article will be made available by the authors, without undue reservation.

## Ethics Statement

The studies involving human participants were reviewed and approved by University of Toronto Research Ethics Board (Protocol # 40567). The patients/participants provided their written informed consent to participate in this study.

## Author Contributions

JJ generated the ideas and framework for the paper, decided which quotes to include, drafted the manuscript, and completed the final editing. AM led the data collection, literature review and writing of the methods section, contributed to drafting, referencing, and editing. Both authors contributed to the article and approved the submitted version.

## Funding

Funding was provided by the Sport Information Resource Centre Match Program to evaluate the Black Female Coach Mentorship Program and the Coaching Association of Canada to develop an anti-racism in coaching literature review. Neither organization had influence in the description of the findings presented here. We acknowledge the support of the Government of Canada's New Frontiers in Research Fund (NFRF), [NFRFE-2018-00651].

## Conflict of Interest

The authors declare that the research was conducted in the absence of any commercial or financial relationships that could be construed as a potential conflict of interest.

## Publisher's Note

All claims expressed in this article are solely those of the authors and do not necessarily represent those of their affiliated organizations, or those of the publisher, the editors and the reviewers. Any product that may be evaluated in this article, or claim that may be made by its manufacturer, is not guaranteed or endorsed by the publisher.
